# Outcome of Primary Stapedotomy in 21 Consecutive Cases of Juvenile Otosclerosis

**DOI:** 10.3390/audiolres14040060

**Published:** 2024-08-19

**Authors:** Valeria Gambacorta, Davide Stivalini, Giacomo Lupinelli, Mario Faralli, Eva Orzan, Giampietro Ricci

**Affiliations:** 1 Department of Medicine and Surgery, Section of Otorhinolaryngology, University of Perugia, 06129 Perugia, Italy; 2 Institute for Maternal and Child Health “Burlo-Garofolo”, 34137 Trieste, Italy

**Keywords:** juvenile, otosclerosis, outcome, stapes surgery

## Abstract

Background/Objectives: Otosclerosis is a relatively uncommon condition that causes conductive hearing loss in children. The preferred treatment for adults is stapedotomy, while for individuals under 18 years old, there is an ongoing discussion about the best treatment approach. Thus, the surgical procedure for the stapes in pediatric patients continues to be a subject of debate. This study aimed to evaluate the results of stapes surgery in children, trying to understand, based on our results, whether this is actually the most suitable option. Methods: The study included 18 patients who underwent surgery between January 2013 and December 2023. The patients’ ages ranged from 11 to 18 years, with an average age of 14.7. Out of the total 21 surgeries, three patients opted for bilateral surgery. Pre- and post-operative data were compared, focusing on the mean air conduction (AC) and bone conduction (BC) thresholds at frequencies of 0.5, 1, 2, and 4 kHz. Additionally, pre-operative thresholds and the post-operative air–bone gap (ABG) were examined. Results: After a year, the air–bone gap was effectively reduced to 10 dB or less in 94% of the 21 cases, and to 20 dB or less in 98% of all cases. Conclusions: Our results and research in the field have consistently shown that stapedotomy, when conducted by skilled otosurgeons, is a reliable and successful procedure for a considerable number of patients. The outcomes it generates are similar to those achieved through the procedure conducted during adulthood.

## 1. Introduction

Otosclerosis was first described by Adam Politzer in 1893 [1]. It is defined as an osteodystrophic disease of the labyrinthine capsule and can cause progressive conductive hearing loss when it occurs at the oval window, leading to the blockage of the stapes plate. Clinical otosclerosis, significantly lower than histological otosclerosis, has a prevalence of around 0.3–0.4% in the Caucasian population and is commonly observed bilaterally [2]. Robinson (1983) found that around 15% of patients with otosclerosis experience conductive hearing loss before the age of 18 [3]. Histological studies conducted by Guild (1944) indicate that otosclerosis occurs in less than 1% of children under 5 years old and in 4% of individuals aged 5 to 18 [4].

It is uncommon for a child to have conductive hearing loss with an intact tympanic membrane. The most frequent causes of this condition include juvenile otosclerosis, congenital fixation of the stapes, tympanosclerosis, fixation of the lateral ossicular chain, or dehiscence of the superior semicircular canal. Diagnosing juvenile otosclerosis may be influenced by factors such as the worsening trend, earlier onset, and familiarity.

Stapedotomy is a widely accepted surgical procedure used to correct conductive hearing loss in adults. In children, the procedure is more complex due to the lower occurrence of the condition, the potential for sensorineural hearing loss after surgery, and the recent introduction of bone anchoring prostheses for treating conductive hearing loss with minimal risks. The insufficient treatment of conductive hearing loss, particularly if it affects both ears, can have significant impacts on language acquisition, cognitive growth, and a child’s educational achievements. There is ongoing debate surrounding the optimal treatment approach, the appropriate age for surgical intervention, and the management of potential complications. Hence, stapes surgery in pediatric patients remains a topic of discussion.

The objective of this study was to assess the outcomes of stapes surgery in pediatric patients, with the aim of determining its suitability as a treatment option.

## 2. Materials and Methods

A total of 18 patients who were operated on between January 2013 and December 2023, ranging in age from 11 to 18 years with an average age of 14.7, were included in the study. Among them, 3 patients with bilateral otosclerosis underwent bilateral surgery, accounting for 16.6% of the total of 21 surgical interventions. Out of the 18 patients, 10 were female (55.5%) and 8 were male (44.4%). The diagnosis of juvenile otosclerosis is made by observing progressive conductive hearing loss that is accompanied by characteristic bone remodeling foci shown on a CT scan. Other potential causes of conductive hearing loss have been identified through otoscopy, the study of audiological data (including tone audiometric examination, tympanograms, and cochleostapedial reflexes), as well as CT images. We did not include cases of conductive hearing loss in our study where there was pre-operative or intraoperative evidence of causes other than juvenile otosclerosis, but we included those with congenital malformations of the ossicular chain. Every patient underwent a comprehensive pre-operative assessment (T0), consisting of a clinical examination, audiometric evaluation (including liminal tone audiometry, tympanograms, cochleostapedial reflexes, and speech audiometry), and a high-resolution computed tomography scan. We conducted a follow-up evaluation of the patients one year later (T1) to assess their long-term outcome. During this assessment, they underwent a comprehensive audiometric evaluation, which included liminal pure tone audiometry, tympanograms, cochleosteal reflexes, and speech audiometry.

All surgical procedures were conducted under general anesthesia using the internal endomeatal route. The neofinestra was formed using a 0.5 mm Skeeter drill, and fluoroplastic prostheses were fitted to the long process of the incus, with a diameter of 0.4 mm and a length ranging from 4 to 4.75 mm.

Comparisons were made between pre- and post-operative data, specifically looking at the mean air conduction (AC) and bone conduction (BC) thresholds at frequencies of 0.5, 1, 2, and 4 kHz, as well as the pre- and post-operative air–bone gap (ABG).

### Statistical Analysis

Means and percentages were calculated. Pre-and post-operative air–bone gaps were compared. The air–bone gaps before and after the surgery were compared using the student’s *t* test. All groups of values exhibit a normal distribution, as determined by the Shapiro–Wilk test. The data was examined using the computer software the jamovi project (2022), version 2.3.

## 3. Results

### 3.1. Surgical Findings

Out of the 21 surgical procedures, 15 were conducted on the right ear, accounting for 71.42% of the total, while the remaining 6 procedures were performed on the left ear (28.57%). In all cases the platen was fixed, and five cases (23.8%) exhibited obliterative otosclerosis. No additional anomalies of the ossicular chain were found. Children with congenital malformations of the ossicular chain were excluded from the study. In one instance, we encountered a floating plate, which presented a complication.

### 3.2. Audiometric Results

**Figure 1:**
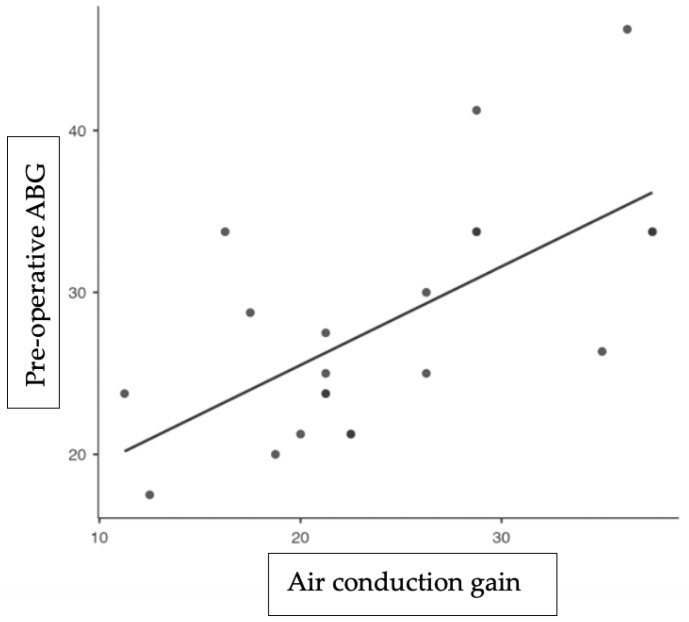
This is a graph illustrating linear regression. Each dot on the graph represents one patient. The *y*-axis represents the pre-operative air–bone gap (in dB), while the *x*-axis represents the post-operative air-conduction gain (in dB). The continuous line illustrates the optimal alignment between the two values, indicating a full restoration of the ABG. As the point moves closer to the continuous line, the ABG shows a more significant recovery.

In the long-term follow-up (T0), at one year for all 21 cases, the air–bone gap was successfully reduced to 10 dB or less in 94% of cases and to 20 dB or less in 98% of all cases. The thresholds for air conduction and ABG showed significant improvement (Figure 1), with average gains of 24.33 dB and 22 dB, respectively. 

There was a statistically significant decrease in the ABG values (*p* < 0.001) between the T0 and T1 groups, as indicated by the Student’s *t* test. The bone conduction threshold experienced an average gain of 0.3 dB (Table 1).

## 4. Discussion

We examined the hearing outcomes of 21 primary stapes surgery procedures in this study. In 94% of cases, the ABG was successfully closed by less than 10 dB; 98% of cases achieved a closure of over 20 dB. Additionally, the average post-operative AC gain was measured at 24.33 dB. In one instance, a floating plate was discovered, necessitating a subsequent surgical procedure six months later.

There is a significant disparity in the number of reports available in the literature regarding the surgical outcomes of stapes surgery in adult patients compared to pediatric patients. In the past, surgical procedures for juvenile otosclerosis were often delayed due to concerns about the potential risk of the sensorineural hearing loss associated with performing stapedotomy at a young age. In a study conducted in 2015, Vincent reported a low incidence of post-operative sensorineural hearing loss. This type of hearing loss is defined as changes in bone conduction thresholds greater than 10 dB. Indeed, the author’s article includes a solitary case at the final follow-up [5]. In 1980, House et al. conducted the first case study of a stapedotomy performed on a patient of pediatric age. The authors initiated the dissertation by addressing a widely held question: “Stapedotomy on a child? Never”. Upon reaching the conclusion, they found that performing a stapedotomy at a young age yielded results that were just as beneficial as those in adults [6]. The thesis has been further supported by subsequent studies confirming the efficacy of stapedotomy, even when performed on individuals in the early stages of life. Carlson et al. (2013), Suk An et al. (2014), Cody Page et al. (2019), and Daniel et al. (2023) have shown through their studies the effectiveness of surgical therapy in restoring a satisfactory hearing threshold in these patients [7,8,9,10]. Specifically, Carlson’s study compared the operative results and outcomes of pediatric patients who underwent primary stapedectomy for congenital stapes footplate fixation (CSFF) or for juvenile otosclerosis. The data from patients who underwent stapedotomy for juvenile otosclerosis revealed that the post-operative ABG was smaller (mean 8.8 dB) compared to patients with congenital stapes fixation (mean 17.2 dB; *p* = 0.04). Additionally, neither group experienced the post-operative onset of profound sensorineural hearing loss [7]. Suk An et al. demonstrated the tendency for improved closure of the air–bone gap in patients with juvenile otosclerosis compared to those with congenital stapes fixation. Their study focused on investigating the causes of stapes fixation in pediatric-aged patients and the outcomes of stapes surgery. The data presented provides evidence for the effectiveness of stapedotomy in individuals diagnosed with juvenile otosclerosis [8]. In their study, Cody Page et al. examined the post-operative results of juvenile otosclerosis, congenital stapes fixation, and tympanosclerosis. They concluded that performing surgery for juvenile otosclerosis in children is a safe procedure. Although the outcomes for juvenile otosclerosis patients were favorable, those with tympanosclerosis experienced only minimal improvement [9].

In a study conducted by De la Cruz et al. (1999), it was found that the ABG closed by less than 10 dB in 82.1% of patients (39 individuals) [11]. Similarly, Lippy et al. (1998) reported a closure rate of 91.7% in 60 procedures [12]. Lescanne et al. (2008) observed a closure rate of 88% in nine procedures [13], while Vincent et al. (2015) reported a closure rate of 93% in 44 procedures [5]. The data align with the findings in our patient cohort, in which and ABG closure below 10 dB was observed in 94% of cases. 

Studies have shown that the rates of improvement in hearing thresholds in adult stapedotomy can range from 78% to 96%. In a study conducted by Simoncelli et al., the outcomes of stapedotomy in adult patients were analyzed. The study found that 80.8% of patients achieved an air–bone gap of less than 10 dB HL (grade A), whereas the total improvement in hearing thresholds was estimated to be 97.3% [14]. These findings align with our research in the area of pediatric stapedotomy in patients with juvenile otosclerosis. Our data analysis has shown that the ABG was decreased by 10 dB in 94% of cases and by less than 20 dB in 98% of cases.

It is noteworthy that juvenile otosclerosis has a higher incidence of obliterating forms compared to adult otosclerosis. Obliterative otosclerosis is defined as the formation of dense new bone in the fossa, which necessitates the use of an oval window drill-out procedure for an abnormally thick footplate. Vincent et al. observed that the obliterate form had an incidence rate of 22%. They also compared this to a previous sample where it was detected in 15% of children and 3% of adults. Regardless, they concur with other writers that although children have a higher incidence of obliterative otosclerosis compared to adults, it should not be considered a reason to avoid stapes surgery in childhood [5]. In their evaluation of the short- and long-term results of stapedotomy in juvenile otosclerosis patients, Lippy et al. discovered a higher incidence of footplate drilling due to obliterative otosclerosis in children (26.7% of cases) compared to adults (10% of cases), which is typically observed. Additionally, they state that while drilling the footplate is more frequently necessary when otosclerosis develops at an early stage, postponing surgery until maturity could potentially exacerbate the footplate pathology and further necessitate drilling. Thus, they also agree that the significant prevalence of juvenile otosclerosis with obliterans form should not be considered a reason to avoid stapedectomy, since delaying the operation may further complicate the procedure [12]. Other previous studies have documented a significant prevalence of obliterative forms in their cohort of patients, with rates ranging from 42% in the study conducted by Cole et al. in 1982 [15] to 47% in the study conducted by Sobolewska et al. in 2018 [2], illustrating the fact that it is a somewhat frequent phenomenon in individuals with juvenile otosclerosis. During our investigation, otosclerosis obliterans was detected in 23.8% of cases. Specifically, it was observed in 5 out of the 21 surgical procedures performed. The higher occurrence of an obliterating form of otosclerosis poses the most significant challenge in performing the surgical operation in pediatric-aged patients. Otherwise, the procedure is similar to that in adults, in whom the obliterating form is less common.

One of the techniques that were suggested by Fisch is the “reversal steps stapedotomy”. This technique involves positioning the prosthesis before removing the stapes superstructure, which can help prevent complications during surgery, such as a floating plate or incus dislocation. Several studies have supported the effectiveness of this technique [2,16,17]. Fish suggested reversing the sequence of steps in the traditional stapedotomy procedure. In accordance with his technique, the dislocation of the incus and the luxation and breaking of the footplate are prevented by first creating a hole in the footplate and securing the prosthesis to the incus prior to removing the stapes superstructure. To gain a clearer understanding of when this type of treatment is appropriate, Malafronte et al. have introduced a new and simplified macroscopic classification of otosclerosis. They categorize it into three types: blue otosclerosis, white otosclerosis, and obliterative otosclerosis. This classification is based on the visual appearance of the footplate prior to removing the stapes superstructure. This classification enables a quick intraoperative determination of the anatomopathological composition of the footplate.

If the visible part of the footplate retains its natural blue color in all areas prior to removing the stapes superstructure, it indicates that the otosclerotic process affecting the oval window rim is limited to the rim of the footplate. This is a case of blue otosclerosis. The white otosclerosis is evident when the visible part of the footplate is predominantly white throughout its entire length; it indicates that the otosclerotic process has spread diffusely throughout most of the footplate.

Otospongiotic focus, when present, masks the visibility of the footplate, resulting in a condition known as obliterative otosclerosis. Fisch recommends employing the technique of reversing steps stapedotomy for both blue and white otosclerosis, however Malafronte et al. emphasize that this procedure should be reserved for blue otosclerosis due to its higher level of safety [16]. The surgeon who performed all the stapedotomies in our patients frequently used the reversal steps stapedotomy technique, particularly when there was convenient access to the round window. Additionally, this was beneficial for mitigating the risk of complications. In our reported case series, 13 out of the 21 surgeries were conducted with reversed surgical timings.

The possible treatment options for juvenile otosclerosis include observation, the use of a traditional prosthesis, the use of a transcutaneous or percutaneous bone-anchored prosthesis, and performing a stapedotomy. Several factors need to be considered when developing an effective therapeutic strategy. These include the severity of hearing loss, whether it affects one or both ears, the patient’s age, the extent of cochlear involvement, the functionality of the eustachian tube, the child’s level of linguistic development, and the impact on their academic progress. Fancello et al. performed a systematic review to elucidate the management of juvenile otosclerosis. The researchers determined that the scarcity of studies on the subject and their lack of consistency prevent final recommendations from being made on this topic at present [18]. Currently, in fact, there are no established treatment protocols for juvenile otosclerosis, nor for the appropriate minimum age for surgery. Interventions conducted on patients at the age of three have been documented in the literature. Weeling et al. were the first individuals to conduct stapedotomy surgery on a child who was younger than 5 years old. In this study, they used a regression analysis to show that the age group at the time of surgery (0–4, 5–9, 10 –14, and 15–18 years) did not have a significant impact on the post-operative air–bone gap result [19]. However, many authors argue that it is important to wait until the young patient is capable of providing informed and conscious consent for the surgical procedure [5,20]. Our case series consisted of patients aged 11 years and older, with an average age of 14.7, aligning with the line of thought of these authors. Although the formal consent, as required by Italian law, in our study was granted in all cases exclusively by the parents, we decided to perform the surgical procedures starting at the age of 11. We believe that at this age, after thorough discussions with both the parents and the patient, we can fully comprehend the patient’s needs and challenges. We believe that achieving this degree of comprehension is not possible with significantly younger patients.

Furthermore, according to Solobewska and Claros (2018), it is not advisable to undergo stapedotomy surgery before the age of five, particularly in cases of unilateral hearing loss and when there are recurrent middle ear infections [2]. Following a recent literature review, it has been suggested that this procedure is both effective and low-risk for patients who are at least 5 years old, do not have recurrent otitis media, and have an ABG > 35 dB [21]. Finally, it is crucial to acknowledge the prognostic markers for the surgical treatment of juvenile otosclerosis, as determined by Kishimoto et al. (2015). These criteria encompass the pre-operative ABG (air–bone gap), pre-operative AC (air conduction), cochlear component of hearing loss, patient’s gender, patient’s age, side of the illness, and radiological evaluation [22].

Computed tomography in particular is essential for both diagnosing and planning treatment. It aids in ruling out other illnesses and pinpointing regions of bone dysplasia. In addition, it can identify potential risk factors such as issues with the facial canal, stapedial persistence, a narrow oval window niche, and other disorders that could result in intraoperative complications or sensorineural hearing loss [2].

## 5. Conclusions

The surgical treatment of juvenile otosclerosis remains a topic of debate in the literature. However, when performed by experienced otosurgeons, stapedotomy has been proven to be a safe and effective technique in a significant number of cases. It yields results comparable to the procedure when carried out in adulthood.

## Figures and Tables

**Table 1:** The table shows the pre- and post-operative audiometric results. AC—air conduction; BC—bone conduction; ABG—air–bone gap; SD—standard deviation; and dB—decibel.

	Pre-Operative (T0)	Post-Operative—One Year (T1)	Difference	*p* Value
AC (mean [SD]) dB	45.53 (6.94)	21.2 (4.65)	24.33 (7.70)	<0.001
BC (mean [SD]) dB	17.5 (5.09)	17.2 (4.36)	0.3 (3.93)	0.732
ABG (mean [SD]) dB	28.2 (7.29)	6.2 (3.05)	22 (7.77)	<0.001

## Data Availability

The data presented in this study are available on request from the corresponding author.
